# Indicators of Psychiatric Disorders in Different Oncology Specialties: A Prevalence Study

**DOI:** 10.1155/2014/350262

**Published:** 2014-04-14

**Authors:** Manuela Polidoro Lima, Flávia L. Osório

**Affiliations:** ^1^Fundação Pio XII, Hospital de Câncer de Barretos, Rua Antenor Antonio Vilela 1331, 14780-000 Barretos, SP, Brazil; ^2^Faculdade de Medicina de Ribeirão Preto, Universidade de São Paulo, 14048-900 Ribeirão Preto, SP, Brazil

## Abstract

*Objective*. This study evaluated the prevalence of various indicators of psychiatric disorders in Brazilian outpatients with cancer and assessed possible associations with sociodemographic indicators. *Materials and Methods*. A total of 1,385 patients were evaluated using the following instruments: Patient Health Questionnaire-4 (PHQ-4), Generalized Anxiety Disorder (GAD-7), Fagerström Test for Nicotine Dependence (FTND), and Fast Alcohol Screening Test (FAST). *Results*. The sample was composed of both genders with a slight predominance of women (55.8%), subjects with incomplete/completed elementary school (59%), married (67.4%), with children (94%), not active from a labor viewpoint (61.6%), and following some type of religion (79.5%). The prevalence of anxiety for the total sample varied between 21.5 and 27.4%. The prevalence of depression was 21.1%, tobacco abuse/dependence was 40.2%, and alcohol was 20.3%. Women had significantly higher levels of anxiety and depression than men. Men had higher levels of substance abuse/dependence indicators than women. *Conclusion*. These results are consistent with the literature, which attests to the high prevalence of psychiatric disorder indicators in cancer patients, especially compared to the general population.

## 1. Introduction

The term “cancer” refers to a set of conditions that have the growth of cells that invade tissues and organs of the human body in common [1]. The causes of cancer are varied, but psychological and behavioral factors such as chronic stress, depression, and social isolation may contribute to the initiation and progression of certain cancers [2, 3].

Conversely, cancer is a risk factor for the development of mental disorders. Symptoms of psychological and physical distress may emerge during cancer treatment in combination with disease systems [4–6].

Attention to the psychosocial aspects of the disease is equally important to cancer treatment [7], especially regarding psychopathologies because they significantly impact morbidity, low adherence to treatment, hospitalization duration, prognosis, quality of life, and patient survival [8–11].

The estimated prevalence of psychiatric disorders in individuals with cancer is approximately 20 to 50% [12–14], and depression exhibits the highest rates [15–18].

Psychiatric disorders and symptoms may represent an adjustment reaction to the disease condition, and these symptoms may also be associated with a general medical condition, such as* delirium* [19]. Clinical complications and metabolic changes, such as hypercalcemia, anemia, vitamin B12 deficiency, and electrolyte imbalance, are also risk factors for mental disorders in cancer patients, especially depression [18].

Therefore, the impact of the cancer and the associated clinical conditions may promote the development of psychiatric disorders, especially mood disorders and psychotic symptoms [20, 21].

Anxiety symptoms are commonly found in cancer patients. Pathological anxiety may be present in physical diseases, medication and drug use, alcohol withdrawal, central nervous system depressants, and the so-called anxiety disorders [22, 23]. The prevalence of anxiety in cancer patients varies between 10 and 30% [24, 25], and it is often associated with the diagnosis stage, procedure performance, and uncertainty about the future [20, 21].

The abuse of psychoactive substances is directly associated with physical diseases, such as cirrhosis, cancer, and cardiovascular disease [26]. The consumption of alcohol and tobacco is a well-established and prominent etiological factor in patients with malignancy in the lungs and esophagus [27].

Various mental disorders are risk factors for the development of some cancers. Mental disorders may appear as comorbidities with the clinical condition, which may negatively impact disease diagnosis and treatment and emotional and financial costs [9–11].

However, oncology clinicians poorly identify mental disorders and their most common symptoms, despite the good responses to pharmacological and/or psychosocial interventions [28–31].

Knowledge of the prevalence of psychiatric disorders in this specific population may increase awareness and identification and mobilize resources for prevention and treatment [6, 32]. Early screening for behavioral and/or psychological/psychiatric disorders in patients seen in health care settings positively affects health care quality and decreases suffering and institutional operational costs [33].

This study evaluated a statistically estimated sample of cancer outpatients at a hospital specializing in cancer according to the presence/absence of major mental symptoms/disorders using simple and quick screening instruments across different oncology specialties.

## 2. Materials and Methods

### 2.1. Subjects

The sample comprised 1,385 subjects. Statistical calculations determined that a minimum sample size of 1,155 subjects was required with an error rate of 3%. The subjects were recruited from a single hospital specializing in cancer, and all subjects were receiving outpatient care (new or return cases) in different oncology specialties. The inclusion criteria were individuals of both genders over 18 years of age. The following exclusion criteria were adopted: severe cognitive impairment as qualitatively evaluated by the applicator and the absence of clinical conditions that would affect responses to the instruments.

### 2.2. Instruments

The following self-assessment scales were used for data collection:

- *Patient Health Questionnaire-4 (PHQ-4)* screens for depression (PHQ-4 D—brief version of PHQ-9) and anxiety (PHQ-4 A—brief version of GAD-7) indicators experienced during the prior two weeks. Pfizer (Pfizer, Inc. Copyright 2005) translated this version into Brazilian Portuguese;
- *Generalized Anxiety Disorder (GAD-7)* screens for typical indicators of anxiety disorders experienced during the prior two weeks. This instrument includes the two items from PHQ-4 A and more five items that enable screening of Generalized Anxiety Disorder. Pfizer (Pfizer, Inc. Copyright 2005) translated this version into Brazilian Portuguese;
- *Fast Alcohol Screening Test (FAST)* evaluates risky, harmful use and alcohol dependence syndrome. This version was translated and validated for Brazilian Portuguese [34];
- *Fagerström Test for Nicotine Dependence (FTND)* measures the degree of physical dependence on nicotine. This version was translated and validated into Brazilian Portuguese [35].
- *Sociodemographic and Clinical Questionnaire* assesses information about sociodemographic data (gender, schooling, civil status, professional situation, and religion) and treatments in mental health.

### 2.3. Data Collection and Analysis

The subjects were selected randomly in reference to the scheduling system of the hospital according to the medical consultation dates. Subjects completed the questionnaires individually, and the evaluator remained available for assistance.

The data were coded according to the ethical (Local Ethics Committee—Process no. 537/2011) and technical recommendations and entered into a database for analysis. The statistical package for social sciences (SPSS) version 19 was used. Descriptive (e.g., mean, standard deviation, and percentage) and parametric statistics (e.g., Student's *t*-test, chi-square, and Pearson correlation test) were used for data analysis. A significance level of *P* < 0.05 was adopted.

## 3. Results

The sample included adult individuals (mean = 50.3; DP = 13.9) of both genders with a slight predominance of women (55.8%). The sample included subjects with incomplete/completed elementary school (59%), married or in a stable relationship (67.4%), with children (86.7%), and not active from a labor viewpoint (61.3%). A total of 79.5% of the sample followed some type of religion, most often Catholicism (68%). Approximately 80% of the sample had no previous psychiatric/psychological history or psychiatric antecedents.

The prevalence of different indicators of psychiatric disorders in the total sample and according to different oncology specialties is shown in Table 1.

Anxiety's symptoms rates between 21.4% and 27.3% were observed for the total group, and Gynecology, Mastology, and Thorax specialties were prominent (Table 1). The prevalence of depression indicators was 18.5% for the total group, and Gynecology, Orthopedics, and Thorax exhibited the highest rates.

The alcohol and tobacco abuse rates were 17.2% and 16.6%, respectively. The percentage prevalence of these abuse rates was higher in the Upper Digestive, Thorax, Head and Neck, and Urology specialties, reaching levels in excess of 30%.

Prevalence indicators for different sociodemographic characteristics of the sample are shown in Tables 2 and 3.

Women had higher anxiety indicators prevalence rates than men, and unmarried subjects exhibited higher anxiety rates than married subjects. This difference according to marital status was observed using the PHQ-4 A instrument, but it was not confirmed using the GAD-7 instrument.

Higher rates of symptoms of depression were associated with females, unmarried, and widowed subjects and subjects who were professionally inactive.

The rates of substance abuse (alcohol and tobacco) were more significant in men, married, and divorced individuals, subjects with less education, subjects who were not professionally active, and subjects who did not follow any religion (Table 3).

The comorbidity rate in the total sample and among different oncology specialties is also noteworthy: 27.1% of the sample exhibited at least one comorbidity, with a prevalence of cooccurrence of anxiety and depression of 11.8%, and 6.6% of the sample had more than two comorbidities.

A total of 43.4% of patients in the Thorax/Lung specialty, 33.5% of the Upper Digestive specialty, and 29.8% of the Urology specialty exhibited indicators of more than one mental disorder.

## 4. Discussion

Cancer and psychiatric symptoms/disorders are strongly associated, and the present study evaluated this association in cancer outpatients undergoing treatment. Screening instruments evaluated symptoms of depression, anxiety, and substance abuse/chemical dependence.

The data showed depression indicators prevalence of 18.5%, which is greater than a previous Brazilian epidemiological study of 9.4% [31] but similar to a meta-analysis that reported a 16.3% depression rate in cancer patients at clinics and cancer hospitals [36]. These data corroborate previous reports of the prevalence of depression in this clinical group.

The prevalence of anxiety indicators varied between 21.4% and 27.3%, which are similar to primary care users (27.9%) as determined using observer-assessment interviews [37]. These data reinforce the important association between physical disease and anxiety, regardless of treatment context.

Moreover, the rate of anxiety indicators in this study is high compared to a previous study that diagnosed 7.6% of cancer patients with an anxiety disorder using a diagnostic interview [38]. However, the choice of instrument may explain this difference, and the present study used screening instruments that measure anxiety symptoms rather than specific disorders.

The prevalence rate of alcohol abuse/dependence for the total sample was 20.3%. This level is higher than patients admitted to general medical specialties (9.8%) and hospitalized cancer patients (15.5%), who were all evaluated using a screening instrument [39].

The tobacco abuse rate for the total sample was 40.2%, which was similar to the prevalence of 44% in patients admitted to gastroenterology and pulmonology wards using screening scales and diagnostic interviews [40]. However, this rate is higher than the Brazilian population (24.1%) [37]. These findings suggest tobacco use as a possible risk factor, especially for oncological diseases of the respiratory system.

The Gynecology, Mastology, and Thorax specialties showed the highest rates of depression and anxiety indicators. One possible explanation for this finding is the exclusive presence of women in the first two specialties. Other evaluation studies of cancer patients reported that women subjects admitted to general hospitals or the general population exhibit high rates of depression and anxiety symptoms [31, 41–45]. It is possible too that, in Thorax specialty, the poorer prognosis can favor these rates [46].

One study evaluated 987 patients with lung cancer and found depressive symptoms in most subjects [47]. Another study evaluated patients with lung cancer using a self-administered Hospital Anxiety and Depression Scale (HADS) and found that 37% had depression symptoms [48]. One possible explanation for this result is suggested by two other studies that showed an increased likelihood of depressive episodes in subjects during nicotine withdrawal, and an even greater likelihood for patients with a history of previous depressive episodes [49, 50]. Therefore, patients with lung cancer may begin a process of reduction or remission of their smoking habit and develop withdrawal symptoms, which promotes the onset of depression and anxiety symptoms.

Men predominated among patients abusing alcohol and tobacco, which has also been demonstrated in previous evaluations in the general population in different countries [39, 51–53]. Therefore, these high rates and the behaviors associated with the male gender, who care less about health and engage in more risky behavior [54–58], support the use of these substances as a risk factor for disease development [27, 59–63]. The higher prevalence rates of alcohol and tobacco abuse in Thorax/Lung and Upper Digestive specialty patients and patients with tumors in the head and neck confirm this hypothesis.

## 5. Conclusions

The data presented are consistent with the previous literature and attest to the high prevalence of indicators of psychiatric disorders in cancer patients, especially compared to the prevalence in the general population. These results support the conclusion that individuals with cancer are more vulnerable to the onset and development of psychiatric disorders.

Conversely, the association between substance abuse and certain cancers indicates that substance abuse can be also a risk factor for disease development.

This study was conducted using a large and statistically estimated sample, but the psychiatric indicators were evaluated using screening instruments. These instruments were validated previously and show adequate sensitivity and specificity, but they only provide the prevalence of indicators but do not indicate disorders per se. The subjects' self-perception also influences these instruments, which may be overestimated or underestimated, and this fact should be considered in data interpretation as well as the fact that all the patients come from a single treatment center.

The high rates indicate the need to screen for psychiatric disorders in cancer patients, especially because of the damaging effects that psychiatric symptoms may exert on patient treatment. Active monitoring and early detection of symptoms may facilitate treatment adherence, decrease the duration of hospital stay, and assist in disease coping.

The study also highlights the need for greater investment in prevention campaigns that discourage the use of legal substances, such as alcohol and nicotine, because these substances are risk factors in cancer development.

## Figures and Tables

**Table 1:** Prevalence of different indicators of psychiatric disorders evaluated using screening instruments.

Specialty (total *N*)	PHQ-4 A		PHQ-4 D		GAD-7		FAST		FTND	
	*N*	%	*N*	%	*N*	%	*N*	%	*N*	%
Head and neck (160)	31	19.4	24	15.0	22	13.8	31	19.4	34	21.4
Upper Digestive (140)	30	21.4	25	18.0	26	18.7	43	30.7	29	20.7
Lower Digestive (120)	31	25.8	23	19.2	22	18.3	24	20.0	21	17.5
Gynecology (146)	63	43.2	44	30.3	47	32.6	17	11.6	20	13.8
Mastology (262)	95	36.4	56	21.3	79	30.3	22	8.4	19	7.3
Melanoma (60)	05	8.3	05	8.3	06	10.0	11	18.3	09	15.3
Neurosurgery (62)	10	16.1	09	14.5	04	6.5	05	8.1	10	16.4
Skin (95)	16	16.8	12	12.6	11	11.7	12	12.6	17	17.9
Thorax/Lung (76)	30	39.5	17	22.4	20	26.3	18	23.7	19	25.0
Urology (236)	63	26.8	35	14.8	54	23.0	52	22.0	48	20.3
Orthopedics (28)	05	17.9	07	25.0	06	21.4	03	10.7	04	14.3

Total (1385)	379	27.36	257	18.55	297	21.44	238	17.18	230	16.60

*N*: frequency, %: percentage, PHQ-4 A: Patient Health Questionnaire-4 A, PHQ-4 D: Patient Health Questionnaire-4 D, GAD-7: Generalized Anxiety Disorder, FAST: Fast Alcohol Screening Test, and FTND: Fagerström Test for Nicotine Dependence.

**Table 2:** Anxiety and depression indicators according to sociodemographic characteristics of the sample (*N* = 1385).

Sociodemographic variables	GAD-7					PHQ-4 D					PHQ-4 A				
	No		Yes		Stat.	No		Yes		Stat.	No		Yes		Stat.
	*N*	%	*N*	%		*N*	%	*N*	%		*N*	%	*N*	%	
Gender															
Male	528	48.8	82	27.6	*χ* ^2^ = 42.40	545	48.4	66	25.7	*χ* ^2^ = 43.8	506	50.4	105	27.7	*χ* ^2^ = 57.4
Female	554	51.2	215	72.4	**P** < 0.001	581	51.6	191	74.3	**P** < 0.001	498	49.6	27	72.3	**P** < 0.001
Did not respond			06					02					02		
Schooling (years)															
≤09 years	632	58.4	183	61.6		668	59.3	150	58.4		598	59.6	220	58.0	
10 to 12 years	280	25.9	73	24.6	*χ* ^2^ = 1.11	279	24.8	74	28.8	*χ* ^2^ = 2.63	254	25.3	101	26.7	*χ* ^2^ = 0.30
>12 years	170	15.7	41	13.8	*P* = 0.57	179	15.9	33	12.8	*P* = 0.26	152	15.1	58	15.3	*P* = 0.85
Did not respond			06					02					02		
Civil status															
Single	173	16.0	51	17.2		171	15.2	53	20.6		145	14.5	79	20.9	
Married/stable relationship	745	68.9	184	62.0	*χ* ^2^ = 7.46	787	70.0	145	56.4	*χ* ^2^ = 18.2	708	70.6	223	58.8	*χ* ^2^ = 18.3
Divorced/separated	90	8.3	38	12.8	*P* = 0.58	93	8.2	35	13.6	**P** < 0.001	82	8.2	47	12.4	**P** < 0.001
Widow/widower	73	6.8	24	8.0		74	6.6	24	9.4		68	6.8	30	7.9	
Did not respond			07					03					03		
Professional situation															
Active	428	39.7	97	33.1	*χ* ^2^ = 4.28	447	39.9	79	31.1	*χ* ^2^ = 6.79	382	38.3	144	38.2	*χ* ^2^ = 0.002
Inactive	649	60.3	196	66.9	*P* = 0.38	673	60.1	175	68.9	**P** = 0.009	615	61.7	233	61.8	*P* = 0.96
Did not respond			15					11					11		
Religion															
Follows	862	79.7	231	78.3	*χ* ^2^ = 0.23	907	80.6	191	74.9	*χ* ^2^ = 4.07	808	80.5	291	77.2	*χ* ^2^ = 1.82
Does not follow	220	20.3	64	21.7	*P* = 0.60	219	19.4	64	25.1	*P* = 0.44	196	19.5	86	22.8	*P* = 0.17
Did not respond			08					04					04		

*N*: frequency, %: percentage, *P*: significance level, *χ* ^2^: chi-square, PHQ-4 A: Patient Health Questionnaire-4 A, PHQ-4 D: Patient Health Questionnaire-4 D, GAD-7: Generalized Anxiety Disorder, and Stat.: statistic.

**Table 3:** Substance abuse indicators according to sociodemographic characteristics of the sample (*N* = 1385).

Sociodemographic variables	FTND					FAST				
	No		Yes		Stat.	No		Yes		Stat.
	*N*	%	*N*	%		*N*	%	*N*	%	
Gender										
Male	268	32.4	342	61.6	*χ* ^2^ = 114	438	38.2	174	73.1	*χ* ^2^ = 97.47
Female	558	67.6	213	38.4	**P** < 0.001	709	61.8	64	26.9	**P** < 0.001
Did not respond			04					0		
Schooling (years)										
≤09 years	391	47.3	423	76.2		657	57.3	161	67.6	
10 to 12 years	265	32.1	90	16.2	*χ* ^2^ = 116	302	26.3	53	22.2	*χ* ^2^=9.97
>12 years	170	20.6	42	7.6	**P** < 0.001	188	16.4	24	10.1	**P** = 0.007
Did not respond			04					0		
Civil status										
Single	138	16.7	84	15.2		184	16.0	40	16.7	
Married/stable relationship	554	67.2	378	68.1	*χ* ^2^ = 3.27	768	67.0	165	69.3	*χ* ^2^ = 7.97
Divorced/separated	70	8.5	59	10.6	*P* = 0.35	103	9.0	26	11.0	**P** = 0.047
Widow/widower	63	7.6	34	6.1		91	8.0	07	3.0	
Did not respond			05					0		
Professional situation										
Active	363	44.3	164	29.7	*χ* ^2^ = 29.5	447	39.2	81	34.2	*χ* ^2^ = 2.13
Inactive	457	55.7	388	70.3	**P** < 0.001	692	60.8	156	65.8	*P* = 0.14
Did not respond			13							
Religion										
Follows	692	84.0	405	73.0	*χ* ^2^ = 24.7	952	83.0	147	62.3	*χ* ^2^ = 51.45
Does not follow	132	16.0	150	27.0	**P** < 0.001	195	17.0	89	37.7	**P** < 0.001
Did not respond			06					0		

*N*: frequency, %: percentage, *P*: significance level, *χ* ^2^: chi square, FAST: Fast Alcohol Test, FTND: Fagerström Test for Nicotine Dependence, and stat.: statistic.
